# Functions and Potential Applications of Circular RNAs in Cancer Stem Cells

**DOI:** 10.3389/fonc.2019.00500

**Published:** 2019-06-13

**Authors:** Ziyang Feng, Shujuan Meng, Hecheng Zhou, Zihao Xu, Ying Tang, Peiyao Li, Changhong Liu, Yongkai Huang, Minghua Wu

**Affiliations:** ^1^Hunan Provincial Tumor Hospital and the Affiliated Tumor Hospital of Xiangya Medical School, Central South University, Changsha, China; ^2^The Key Laboratory of Carcinogenesis of the Chinese Ministry of Health, The Key Laboratory of Carcinogenesis and Cancer Invasion of the Chinese Ministry of Education, Cancer Research Institute, Central South University, Changsha, China; ^3^The Xiangya Hospital, Central South University, Changsha, China; ^4^The Affiliated Zhuzhou Hospital Xiangya Medical College, Central South University, Zhuzhou, China

**Keywords:** circRNAs, cancer stem cell, biomarker, vaccine, therapy resistance

## Abstract

Circular RNAs (circRNAs) were discovered in the 1970s, but they have drawn increasing attention in recent years. Currently, we know that circRNAs are not “wrongly spliced” during transcription but play important roles in the initiation and development of various diseases, including cancers. Recently, a growing number of studies have suggested that cancer stem cells (CSCs) may contribute to the origination and maintenance of cancers. This review briefly introduces the major functions of circRNAs, including interacting with other noncoding RNAs, competing with pre-mRNA splicing, binding with proteins to form a scaffold, promoting protein nuclear translocation and even translating proteins in a cap-independent manner. Furthermore, we describe the regulatory mechanism of circRNAs in CSC phenotypes and discuss the potential clinical applications of circRNAs in CSC-targeted therapy, including functioning as new biomarkers, acting as vaccines and breaking the therapeutic resistance of CSCs. Finally, we discuss the major limitations and challenges in the field, which will be beneficial for the future clinical use of circRNAs.

## Background

Cancer stem cells, a special subpopulation of cancer cells, exhibit self-renewal properties as well as high proliferation and multidirectional differentiation potential. According to current studies, the main origins of CSCs include (1) adult stem cells (1), (2) tumor cells (2), (3) differentiated cells (3), and (4) cell fusion (4). Through asymmetric cell division, a CSC can give rise to a new CSC and a new differentiated daughter cell. As a result, CSCs are able to maintain their number and play key roles in the generation and development of cancers, such as leukemia, glioma, head and neck cancer, lung cancer, breast cancer, and hepatocellular carcinoma. CSCs are clinically important since they are considered the root cause of cancer recurrence and therapy resistance.

CircRNAs, a kind of special endogenous noncoding RNA (ncRNA), are famous for their covalently closed loop structures without 5′ caps or 3′ poly A tails (5, 6). With the development of high-speed RNA sequencing technologies, numerous circRNAs have been identified. In contrast to linear RNAs, circRNAs are more stable because of their circular structures, which can help them escape degradation by enzymes. Recent studies have shown that circRNAs can function as potential diagnostic and prognostic biomarkers and as therapeutic targets in various diseases, especially in cancer, and may be involved in the regulation of CSCs. We have described the categories, biogenesis, and functions of circRNAs (5) and the potential role of circRNAs in tumor immunity regulation and immunotherapy (6). In this review, we provide an advanced understanding of the function of circRNAs and discuss their potential application with respect to CSCs.

## Advances in Understanding CircRNA Functions

### Interactions Between CircRNAs and Other ncRNAs

**Figure 1:**
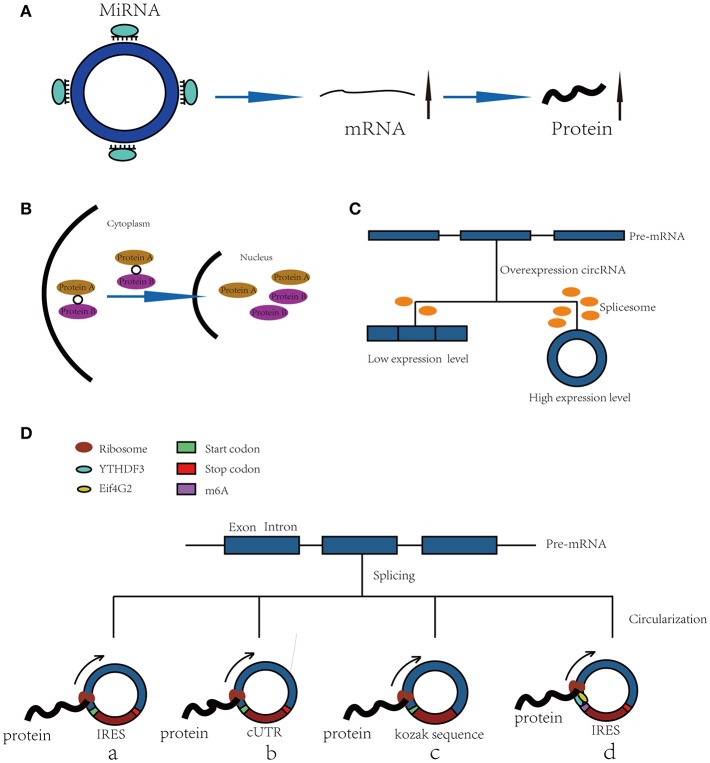
Functions of CircRNAs. **(A)** CircRNAs function as miRNA sponges to increase expression of target gene. **(B)** CircRNAs bind with proteins and promote protein nuclear translocation. **(C)** Circularization of exons can compete with pre-mRNA splicing. **(D)** CircRNAs function as templates of translation: (a) Internal Ribosomal Entry Site (IRES)-dependent mechanism; (b) UTRs of ribo-circRNAs (cUTRs)-dependent mechanism; (c) Rolling circle amplification (RCA) mechanism: the circRNAs include a kozak sequence and start codon but without stop codon (d) M6A modification of circRNAs accelerates the translation of circRNAs.

“MicroRNA sponge” is one of the most classic functions of circRNAs in post-transcriptional regulation. CircRNAs can act as “sponges” to bind microRNAs (miRNAs) and regulate the expression of target mRNAs (7, 8). CRD1as is a circularized and highly conserved single-exon circRNA in the mammalian brain (9). It contains 63 conserved binding sites for miR-7 and can repress the function of miR-7 and increase the expression levels of TGFBR2 (10), SMAD (11), PARP (12), and other genes. CircHIPK3 is formed from HIPK3 exon 2 and can bind with miR-124; thus, the silencing of circHIPK3 can inhibit human cell proliferation by downregulating target genes of miR-124, such as IL6R and DLX2 (13). In addition, circHIPK2 (14), circBIRC6 (15) and circ-SRY (7) can also bind with miRNAs to regulate mRNA expression (Figure 1A).

In addition, the interactions among circRNAs, long noncoding RNAs (lncRNAs) and miRNAs are also drawing increasing attention. Aruo Nan et al. found that lncRpa and circRar1 induced the upregulation of the apoptosis-associated factors caspase8 and p38 at the mRNA and protein levels via direct and specific binding with miR-671. Furthermore, their study suggested a positive regulatory relationship between lncRpa and circRar1, although the mechanism is not clear (16). This study was the first validation of the interactions among lncRNA, circRNA, and miRNA to mediate lead-induced neuronal cell apoptosis, which suggested a complex regulatory network between circRNAs and other ncRNAs.

### Forming a Scaffold and Promoting the Nuclear Translocation of Proteins

CircRNAs can bind with proteins to act as a scaffold to affect their functions or promote their nuclear translocation. For example, circFoxo3 can act as a scaffold to bind with the cell cycle proteins cyclin-dependent kinase 2 (CDK2) and cyclin-dependent kinase inhibitor 1 (p21) to form a ternary complex. Generally, CDK2 interacts with cyclin A and cyclin E to facilitate cell cycle entry, but when circFoxo3, CDK2 and p21 form a complex, CDK2 cannot interact with cyclins A and E, thus blocking cell cycle progression (17). In addition, in p53 wild-type breast cancer cells, P53 can form a complex with H2AX and circ-Ccnb1. In p53 mutant cells, mutated P53 cannot bind with H2AX, but circ-Ccnb1 can function as a scaffold to enhance the interaction between H2AX and Bclaf1. Bclaf1 can function as a tumor repressor by binding with H2AX, thereby inducing cell death (18, 19).

Furthermore, a recent study has shown that circRNAs also bind with proteins and promote their nuclear translocation. Circ-DNMT1 can bind with P53 and AUF1 and enhance their nuclear translocation; the nuclear translocation of p53 can induce cellular autophagy, and the nuclear translocation of AUF1 increases the stability of Dnmt1 mRNA and enhances breast cancer cell proliferation (20). The molecular simulation results predicted the minimal binding region of circ-Dnmt1 for p53 to be “AACCTTCAC” “AAC”“AGGAAGAA” and the minimal binding site of circ-Dnmt1 for Auf1 to be “AACCTTCAC” “TTACA” “C” “C” “GAGT” “AG”“AGAA” (20). In addition, circ-AMOL1 can interact with STAT3 and promote its nuclear translocation, and STAT3 can increase the transcription of DNMT3a and promote the methylation of the miR-17 promoter, which was found to induce injury repair in a mouse excisional wound model (21). The molecular simulation also predicted the minimal binding region of circ-Amotl1 for Stat3 to be “AACCTTCAC” “AAC” “AGGAAGAA” (21). This binding region is the same as that of circ-Dnmt1 for p53, suggesting that this region is an important sequence for circRNA binding with proteins (Figure 1B).

### Exon Circularization Competes With Pre-mRNA Splicing

The spliceosome plays important roles in both circRNA production and pre-mRNA splicing, meaning that the spliceosome is a key factor in the competition of exon circularization and the canonical splicing of pre-mRNA (22). Ashwal-Fluss et al. mutated the 5′ splice sites from GU to CA flanking the PVT1 or CRKL exons, which resulted in a strong decrease in the generation of circRNAs, suggesting that exon circularization is dependent on the spliceosome (or at least U1). Therefore, increasing the efficiency of linear splicing can significantly repress the generation of circRNAs (22, 23). Similarly, muscleblind (MBL) can bind with flanking introns and promote the circularization of circMbl. Overexpression of MBL was found to increase the expression of circMbl by 13-fold, but linear Mbl was decreased by 2-fold because some of the spliceosomes are occupied by circRNAs, providing new insight into the transcriptomic regulation of circRNAs (Figure 1C).

### Translating Proteins in a Cap-Independent Manner

Because circRNAs do not have a 5′ cap, they are considered to be ncRNAs without the capacity to translate proteins. However, increasing numbers of studies have shown that circRNAs can function as templates for RNA translation. At present, there are four kinds of mechanisms for the translation of proteins from circRNAs (Figure 1D).

#### Internal Ribosomal Entry Site (IRES)-Dependent Mechanism

Internal ribosomal entry sites (IRESs) are special sequences that can directly induce the binding of initiation factors and ribosomes to RNAs without the help of a 5′-cap structure, playing an important role in the translation of circRNAs (24–28). For example, there is a start codon, a stop codon and a 753-nucleotide open reading frame (ORF) in circZNF609, and phylogenetic analysis has shown that the 5′ UTR in circZNF609 is more conserved than the UTRs of linear ZNF609 mRNA, which enable them to function as IRESs and initiate the translation of circRNA (27). This study shows that by inserting a 3 × FLAG coding sequence upstream of the STOP codon, circZNF609 can be translated to a fusion protein with FLAG sequences in a cap-independent manner both *in vitro* and *in vivo*. Another example is circ-SHPRH, which is generated from exons 26–29 of the SHPRH gene and contains an IRES sequence and an ORF that can be translated into a 146-amino-acid protein. Interestingly, circ-SHPRH uses the overlapping genetic codes “UGAUGA” to generate a “UGA” stop codon and an “AUG” start codon. Thus, cir-SHPRH is the first endogenous circRNA known to use overlapping initiation and termination codons to translate a protein, which indicates the complexity of protein translation from circRNAs (28).

#### UTR of ribo-circRNA (cUTR)-Dependent Mechanism

In 2017, a study identified a group of special circRNAs called ribo-circRNAs (29). These circRNAs contain at least one ribosome footprinting read according to previously published ribosome footprinting (RFP) datasets and can be predominantly bound to membrane-associated ribosomes (29). These circRNAs have the same start codons as the corresponding linear mRNAs and can be translated into proteins with the help of cUTRs. For example, circMBL is a ribo-circRNA: researchers generated a circRNA reporter that contains a Renilla luciferase ORF downstream of the circMbl cUTR and found that it is capable of driving Renilla translation. The translation of circRNA was not affected by the inhibition of cap-dependent translation with 4E-BP protein. However, the translation efficiency of cUTRs is weaker than that of CrPV IRES, which means that the IRES activity of the special sequence is still controversial. Interestingly, another circRNA reporter with the circMBL cUTR in a reverse orientation can also drive translation, suggesting that the functional element is structural in nature.

#### Rolling Circle Amplification (RCA) Mechanism

In addition to IRESs and cUTRs, circRNAs can also be translated into proteins in a manner similar to the rolling circle amplification (RCA) mechanism (24), even without any IRES sequence, poly-A or cap structure. Researchers prepared artificial circular RNAs with an infinite open reading frame and tested their translation in eukaryotic systems. These artificial circRNAs included the Kozak sequence, which plays a major role in the initiation of translation of eukaryotic systems, and several FLAG sequences but lacked a stop codon. The results showed that FLAG-containing peptides of high molecular weight were detected when these circular RNAs were introduced into HeLa cells. In addition, the results suggested that the translation initiation on these circular RNAs is unlikely to be attributable to the IRES activity of FLAG–coding sequences. No translation product can be observed when a stop codon is inserted into the circRNAs, meaning that a finite ORF is not beneficial to RCA mechanism-dependent translation.

#### M6A Modification of RNA Accelerates the Translation of CircRNAs

Recently, N6-methyladenosine, the most abundant modification in RNA, was found to play an important role in the translation of circRNAs (30). Researchers have shown that by inserting m6A motifs before the start codon of a circRNA reporter and transfecting them into HEK293 cells, these circRNAs can be efficiently translated into proteins. This capacity is so strong that a single m6A site is sufficient to start translation. The M6A reader YTHDF3 can recruit Eif4G2, two proteins that are key regulatory factors in translation initiation; their depletion can greatly decrease the translation of circRNAs but has no influence on mRNA. Even in circRNAs, the m6A modification is widespread, and many such modified circRNAs are associated with polysomes, which suggest that the translation of circRNAs might be common in the human transcriptome.

## CircRNAs are Involved in the CSC Phenotype

### Self-Renewal

Because of their self-renewal capacities, CSCs play important roles in oncogenesis. As a newly recognized kind of functional RNA, circRNAs are also involved in the self-renewal of CSCs. CircGprc5a, which is upregulated in bladder cancer and bladder CSCs, can be translated into a new short peptide. This peptide can bind with Gprc5a and increase its function to drive the self-renewal of bladder CSCs (31). Additionally, circ-ITCH can function as a sponge of miR-214, and miR-214 can promote the self-renewal and stemness of CSCs by repressing the expression of Wnt-Regulatory Protein CTNNBIP1 (32). These studies suggest that circRNAs play important roles in regulating the self-renewal of CSCs.

### Proliferation, Differentiation, and Apoptosis

Hsa_circ_0020397 can bind with miR-138 and regulate the expression of telomerase reverse transcriptase (TERT) (33). TERT is a crucial regulatory protein that promotes proliferation by augmenting tRNA expression in CSCs (34).

CircRNAs also participate in the differentiation of normal stem cells and cancer stem cells. For example, circZNF91 is abundantly expressed and upregulated during human epidermal stem cell differentiation (35). In addition, hsa_circ_0005075 can function as a sponge of miR-93 (36), and miR-93 can completely block CSC differentiation by promoting the mesenchymal-epithelial transition (MET) and downregulating the TGFβ signaling pathway and related stem genes, such as AKT3, SOX4, and STAT3 (37).

Furthermore, circRNAs are also involved in the apoptosis of CSCs. CircUBAP2 can function as a sponge of miR-143, thus enhancing the expression and function of anti-apoptotic Bcl-2, which is an important anti-apoptosis molecule in CSCs (38, 39). Cirs-7 can facilitate the cytoplasmic localization of NF-κB, which inhibits the activity of the ubiquitin C-terminal hydrolase L1 (UCHL1) promoter. Thus, cirs-7 can upregulate UCHL1 expression, and UCHL1 plays important roles in promoting the degradation of b-site APP-cleaving enzyme 1 (BACAE1) (40). BACAE1 can participate in the apoptosis of ovarian cancer stem cells (OCSCs) by elevating the levels of APP and the Aβ1-42 peptide (41). Additionally, the circRNA_15698/miR-185/TGF-β1 axis promotes extracellular matrix (ECM)-related protein synthesis and induces ECM accumulation (42), which suggests that circRNAs have high potential to change the surrounding microenvironment and induce anoikis of CSCs, which is a type of apoptosis induced by a lack of extracellular matrix attachment (43).

### Migration and Invasion

Circ-008913 is involved in the acquisition of CSC-like properties and induced the migration and invasion in arsenite-transformed HaCaT cells (T-HaCaT cells) (44). Another study showed that arsenite can decrease the expression of circ008913, which functions as a competing endogenous RNA for miR-889 and decreases the expression of its direct target gene, DAB2IP (45, 46). A decrease in DAB2IP can promote the expression of zinc finger E-box-binding homeobox1 (ZEB1) by regulating the PI3K-Akt-mTOR signaling pathway, thus increasing the mRNA levels of stem cell markers and playing important roles in the maintenance of CSC-like properties (46, 47). In addition, hg19_circ_0005033 is one of the most prominent circRNAs in CD133+CD44+ laryngeal cancer stem cells (LCSCS) (48). It functions as a ceRNA to increase the expression of STAT5A, which induces stem-like cell properties and the epithelial-to-mesenchymal transition and thus promotes the migration and invasion of CD133+CD44+ LCSCs (48, 49).

As mentioned above, several circRNAs have been found to play important roles in the regulation of CSC phenotypes (Table 1), but most of the available studies are related to their intracellular functions. In the progression and phenotype regulation of CSCs, the communication between CSCs and their surrounding microenvironment is also quite important (50), but related studies about circRNAs are rare. Recently, Xiangyu Da et al. found that circRNA_100284 secreted from exosomes of arsenite-transformed human hepatic epithelial (L-02) cells can be transferred into normal L-02 cells and is involved in the malignant transformation of L-02 cells through circRNA_100284/mir-217/EZH2 axis (51). Furthermore, circRNAs can be transferred to exosomes to influence the tumor microenvironment and can function as a potential biomarker for human illness (52, 53). These studies indicate that circRNAs can indeed be secreted into exosomes and are involved in cell-cell communication, suggesting that studies about circRNAs should not be limited to the intracellular level. More studies on the communication between circRNAs and CSCs microenvironment should be performed.

**Table 1:** Potential functions of circRNAs in cancer stem cells.

**CircRNAs**	**Host gene**	**Function**	**Target gene**	**Related CSCs**	**Potential effect**
CircGprc5a (31)	Gprc5a	Translating	–	Bladder CSCs	Driving the self-renewal of bladder CSCs (31)
Circ-ITCH (32)	ITCH	miRNA sponge (miR-214)	CTNNBIP1	Cancer stem-like cells in lung adenocarcinomas.	Suppressing self-renewal and stemness of CSCs (32)
Hsa_circ_0020397 (33)	DOCK1	miRNA sponge (miR-138)	TERT	Liver cancer stem cells	Promoting malignant proliferation of CSCs (34)
hsa_circ_0005075 (36)	EIF4G3	miRNA sponge (miR-93)	AKT3, SOX4 and STAT3	Breast cancer stem cells	Promoting proliferation and differentiation of CSCs (37)
CircUBAP2 (38, 39)	UBAP2	miRNA sponge (miR-143)	Bcl-2	Colon cancer stem cells	Suppressing apoptosis of CSCs (38, 39)
Cirs-7 (40)	CDR1 NAT	Facilitate cytoplasm localization of NF-κB	–	Ovarian cancer stem cells	Participating in apoptosis of CSCs (41)
Circ-008913 (44–47)	ADAT1	miRNA sponge (miR-889)	DAB2IP	–	Regulating acquisition of CSC-like properties and neoplastic capacity of arsenite-transformed HaCaT cells (44–47)
Hg19_circ_0005033 (48, 49)	–	miRNA sponge (miR-45121)	STAT5A	CD133+ CD44+ laryngeal cancer stem cells	Promoting proliferation, migration, invasion, and chemotherapy resistance of CSCs (48, 49)

## Potential Application Prospects of CircRNAs in CSC-Targeted Therapy

### CircRNAs May Act as New Markers of CSCs

Some CSCs are derived from normal stem cells (1, 54). Thus, the similarity between CSC markers and normal stem cell markers results in risks and limitations of CSC-targeted therapy, as normal stem cells will be affected when targeting these markers. For example, CD44 functions as a biomarker and a therapeutic target in CSCs (55). However, CD44 is also a marker of normal stem cells, meaning that normal stem cells can also be affected by targeting CD44 in CSC therapy (56). Thus, new biomarkers for CSCs and normal stem cells are becoming an urgent need.

**Figure 2:**
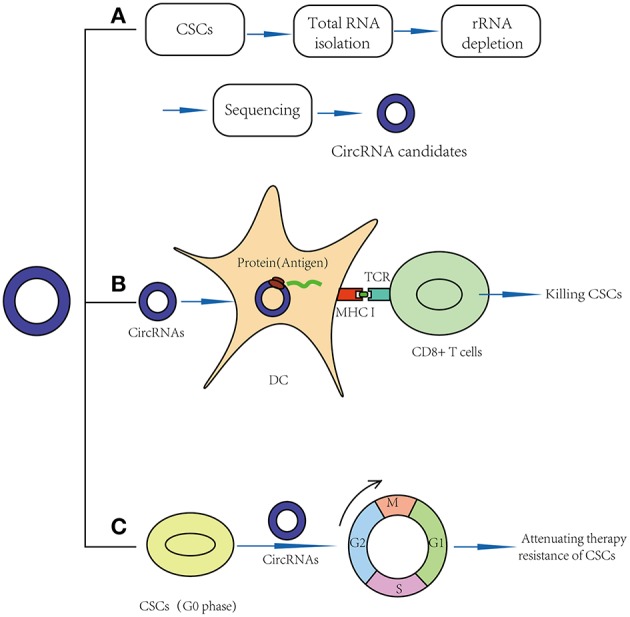
Application prospects of circRNAs in CSCs targeted therapy. **(A)** CircRNAs may act as new markers of CSCs. **(B)** CircRNAs may act as vaccines in CSC targeted therapy: circRNAs may be transfected into dendritic cell and translate proteins, thus function as potential antigens and activate CD8+ T cells in CSC-based vaccines. **(C)** CircRNAs may break quiescence and therapy resistance of CSCs.

Some laboratory studies have shown that circRNAs may function as new potential markers in normal stem cells and CSCs (Table 2; Figure 2A). For example, circASXL1 and circHIPK3 have been found to be highly expressed in human mesenchymal stem cells and can function as potential biomarkers (60). Twenty-seven circRNAs were found to be aberrantly expressed in BCSCs, such as chr1:151630710|151641111, chr12:116534473|116549317 and chr12:69210591|69218431 (57). In addition, CD133+CD44+ LCSCs showed different expression levels of 3684 circRNAs, such as hg19_circ_0003081, hg19_circ_0008472, and hg19_circ_0005033 (48). As mentioned above, circGprc5a is also an upregulated circRNA in bladder CSCs (31). These circRNAs are new candidates for CSC biomarkers, but their expression levels in other CSCs have never been detected, meaning that they might be aberrantly expressed in other CSCs. More studies on these circRNAs should be performed.

**Table 2:** Application prospect of circRNAs in CSCs targeted therapy.

**CircRNAs**	**Parental gene**	**Related CSCs**	**Potential application**
chr1:151630710|151641111 (57)	SNX27	BCSCs	New biomarkers
chr12:116534473|116549317 (57)	MED13L	BCSCs	New biomarkers
chr12:69210591|69218431 (57)	MDM2	BCSCs	New biomarkers
hg19_circ_0003081 (48)	MFF	LCSCs	New biomarkers
hg19_circ_0008472 (48)	ATXN10	LCSCs	New biomarkers
hg19_circ_0005033 (48)	HIBADH	LCSCs	New biomarkers
circGprc5a (31)	Gprc5a	Bladder CSCs	New biomarkers and antigen
circLMO7 (58)	LMO7	–	Break therapy resistance
cia-cGAS (59)	CGAS	Hematopoietic system CSCs	Break therapy resistance

### CircRNAs May Act as Vaccines in CSC-Targeted Therapy

CSC-based vaccines include cell-based vaccines, DNA-based vaccines and mRNA-based vaccines, which have been shown to play important roles in CSC-targeted therapy (61). However, there are still several limitations: (1) The half-life of mRNA-based vaccines is <24 h, which means decreased stability (62). (2) DNA-based vaccines require DNA integration into the genome, which may lead to mutations (63, 64). (3) Total RNA vaccines or cell-based vaccines may induce autoimmunity (64).

Recently, clinical studies have shown that mRNA can be transfected into dendritic cells (DCs) and translated into proteins to function as antigens (65), meaning that circRNAs, especially those that can be translated into proteins, may function as potential antigens and activate CD8+ T cells in CSC-based vaccines (Figure 2B). For example, in 2010, Jian-Cong Sun found that mature DCs loaded with CD133+ hepatocellular carcinoma stem cell RNA could induce a CD8+ cytotoxic T lymphocyte response against hepatocellular carcinoma stem cells *in vitro* (66). In 2011, an experimental study showed that DCs transfected with 9L glioma stem-like cell RNA can significantly inhibit glioma growth and prolong the survival of 9L glioma-bearing rats (67). In 2013, a clinical trial (NCT00846456) suggested that amplified mRNAs from glioma CSCs can be transfected into autologous DCs, and these DCs can then be injected intradermally to act as vaccines. In this study, compared to the control group, progression-free survival was 2.9 times longer in vaccinated patients. No patients developed adverse autoimmune events or other side effects (68).

Similarly, we believe that circRNAs might also play important roles by acting as vaccines in CSC-targeted therapy (Table 2). For example, the results of a functional study suggested that circGprc5a is upregulated in bladder tumors and CSCs and positively regulates the activity of bladder CSCs. CircGprc5a showed strong coding potential. Interestingly, this peptide can bind with Gprc5a protein, a surface protein highly expressed in bladder CSCs (31). This result suggested that this peptide might bind with Gprc5a surface protein and function as a new surface antigen in bladder CSC vaccines. Compared with traditional vaccines, circRNAs might have several merits: (1) Compared with the traditional “mRNA vaccine,” circRNAs have a longer half-life period because of their special circular structure (69). Thus, circRNAs may be translated continually into proteins for a longer time, and a small amount of circRNAs may suffice to sensitize DC cells. (2) Similar to mRNA vaccines, circRNAs can be translated into proteins in the cytoplasm; thus, they do not need to integrate into the genome and are safe. (3) In addition to endogenous circRNAs, we can also try to design artificial circRNAs, which could express purified CSC-associated antigens and transfect them into DC cells. Compared to total RNA vaccines or cell-based vaccines, the purified antigen could reduce the risk of autoimmunity (64).

Although there are many advantages to circRNAs functioning as new vaccines, circRNA studies are in the initial stage; thus, all related clinical studies have focused on mRNA vaccines. Furthermore, the *in vitro* synthesis of circRNAs is currently still performed with recombinant enzymes, which would be costly if circRNAs are required for vaccines (70). Thus, the potential application of circRNAs as vaccines has still not been proven in clinical trials. This new field is waiting to be explored.

### CircRNAs May Attenuate the Therapeutic Resistance of CSCs

The therapeutic resistance of CSCs is the root cause of tumor recurrence. Quiescence, a property that keeps a cell in a nondividing state (G0 phase) but allows the cell to re-enter the cell cycle at a later time, is important in the therapeutic resistance of CSCs, especially in haematopoietic system tumors. For example, breaking quiescence and enhancing the proliferative phenotype of AML stem cells with granulocyte colony-stimulating factor (G-CSF) led to increased sensitivity to the chemotherapeutic agent cytarabine (71). In addition, ablation of the F-box protein Fbxw7 in Phi + leukemia CSCs leads to decreases in c-Myc, Notch, and cyclin E and re-entry into the cell cycle and increases the sensitivity to imatinib (72).

Recently, some functional studies have shown that circRNAs were also found to be involved in stem cell quiescence (Table 2). For instance, circLMO7 acts as a competing endogenous RNA for miR-378a-3p. Thus, circLMO7 can increase the number of myoblasts (a kind of unipotent stem cell) in the S-phase of the cell cycle and decrease the proportion of cells in the G0/G1 phase by repressing the function of miR-378a-3p (58). In addition, a circular RNA named cia-cGAS was highly expressed in the nuclei of long-term haematopoietic stem cells (LT-HSCs) (59), whose deficiency in mice can lead to a dramatic decrease in dormant LT-HSCs in the bone marrow. Cia-cGAS can bind cGAS with stronger affinity than its self-genomic DNA, which prevents cGAS from recognizing self-DNA and consequently suppresses the cGAS-mediated production of type I IFNs in LT-HSCs. The decrease in type I IFNs plays important roles in maintaining a number of dormant LT-HSCs. Moreover, type I IFNs play an important role in CSC activation, which means that cia-cGAS may function as a potential therapeutic target to inhibit the quiescence and dormancy of CSCs through the cia-Cgas/Cgas/I IFN axis (59, 73), especially in haematopoietic system tumors. By breaking quiescence, circRNAs play crucial roles in decreasing the numbers of CSCs and increasing their sensitivity to drugs, providing a new insight for CSC therapy (Figure 2C).

However, some problems remain. For example, circLMO7 and cia-cGAS have also been found to be expressed in normal stem cells, which means that targeting these circRNAs might have potential risk during the treatment (58, 59). In addition, circLMO7 and cia-cGAS can break quiescence in stem cells, but the delivery of circRNA is still a critical issue. For example, many previous studies encapsulated nucleotides into ~100-nm nanoparticles. However, such nanoparticles are easily accumulated in liver and spleen. Furthermore, some cancers have inherent barriers that can prevent the penetration of such nanoparticles, such as pancreas and brain tumors (74, 75). Thus, how to send these circRNAs accurately into targeted CSCs is still a problem waiting to be solved.

## Current Challenges and Related Technologies

Recent studies have shown that circRNAs might seem to be valuable in disease diagnosis and treatment, especially in cancer. However, there are several obstacles for circRNA research that are typically overlooked and require further study: (1) Claiming a treatment role of circRNAs in CSCs is somewhat risky because there are still not any direct clinical studies focusing on CSC-specific circRNA treatment. (2) It is difficult to deliver circRNAs efficiently to CSCs with a long-term sustained effect and without immunological rejection. Generally, individual nucleotides can enter cells freely, but circRNAs and circRNA-generating vectors are always insufficiently hydrophobic and too large (>1,000 Daltons) to passively pass the cell membrane's phospholipid bilayer (70, 76, 77). (3) Although siRNA and shRNA have been widely used in the silencing of circRNA (78), it is important to note that siRNA and shRNA molecules have been designed to target the back splice site of circRNA, but the partial complementarity of a half-RNAi sequence (~10 nt) to its parent linear RNA may still have an effect on the expression of the parent gene (79, 80). Furthermore, the off-target effect is always unavoidable (79). Thus, how to suppress circRNAs without an influence on parent gene expression is still a question. (4) CircRNAs are not sufficient as specific biomarkers for CSCs. Although some studies have reported that certain circRNAs are specific for certain CSCs, their expression levels in other CSCs are still unknown. However, the detection of CSCs relies on a combination of several types of data, meaning that circRNAs can still play important roles when combined with other biomarkers.

For the above challenges, several related technologies need to be further applied and studied: (1) circRNA profiling and identification technologies: The identification of a circRNA is the most important step before studying it. CircRNA-seq and microarray are two widely used technologies for the genome-wide profiling of circRNAs (81, 82). The principle of circRNA-seq and microarray is similar to that of linear RNA, but the issue is how to deplete linear RNAs and enrich circRNAs. After extracting total RNA, DNA can usually be removed with DNase treatment, and linear RNAs can be removed with RNase R treatment to further identify the presence of circRNAs. Besides, PCR with “divergent primers” and Sanger sequencing to confirm “back splice sites” is the most classical method to identify circRNAs after sequencing or microarray. (2) CircRNA delivery technologies: Recent studies have shown that extracellular vesicles (EVs) can function as delivery vectors for therapeutic interventions (83, 84). Furthermore, circRNAs were also found to be enriched in EVs and can be transported from cell to cell (51). Therefore, it is possible that EVs, including exosomes, could be engineered to deliver circRNAs efficiently to a target tissue (85). Further more, a recent study has found that small nucleic acid drugs can be delivered to targeted cells with charge-matched Y-shaped block catiomers (75). Engineered Y-shaped block catiomers can bind tightly with nucleic acid drugs in the bloodstream through selective ion-pairing and to generate a dynamically equilibrated unit polyion complex (uPIC). These uPICs are able to enter both stroma-rich pancreatic tumors and brain tumor tissues and exert significant antitumour activity. (3) Anti-sense oligonucleotides (ASOs) and clustered regularly interspaced short palindromic repeats-associated nuclease Cas9 (CRISPR/Cas) technology: With the exception of back splice sites, ASOs could also be designed to target intron sequences, binding sites for transacting splicing factors or the flanking intronic Alu repeats of circRNAs (86–88). Compared with siRNA and shRNA, there are more targeted sites for ASOs, and off-target effects are easier to avoid. Furthermore, CRISPR/Cas9 can function as a highly efficient tool to knock out circRNAs. Early studies have shown that flanking intronic complementary sequences play important roles in the production of circRNAs (89). Thus, removal of the flanking intronic complementary sequences by CRISPR/Cas9 can partially and even completely suppress expression of circRNAs without affecting the linear mRNA (13, 90). But more related studies need to be performed, such as building a database of circRNA expression in CSCs, along with related clinical trials. Thus, the practical application of circRNAs in clinics remains nascent and warrants further intensive investigation.

## Conclusion

Research regarding the regulatory roles of circRNAs on CSCs is still in the initial stages. Although the existing research has indicated that the aberrant expression of circRNAs plays a key role in the regulation and progression of cancers and CSCs, some problems remain to be solved. It is important to note that current studies about circRNAs are mainly focused on the intracellular level, while few studies have explored how circRNAs change in the CSC environment, which might be a new direction for future studies. In addition to the regulatory effect of circRNAs on CSCs, they can also act as tumor biomarkers (5), affect tumor immune regulation (6), and influence the cell microenvironment (91, 92). Hence, circRNAs may be a new target and can provide new insights and directions for the treatment of cancer and other diseases.

## Author Contributions

ZF wrote and revised the manuscript, and was the major contributor. SM, HZ, ZX, YT, PL and CL collected the related paper. MW and YH participated in the design of the review and helped to draft and revised the manuscript. All authors read and approved the final manuscript.

### Conflict of Interest Statement

The authors declare that the research was conducted in the absence of any commercial or financial relationships that could be construed as a potential conflict of interest.

## Abbreviations

circRNAs: Circular RNAs
lincRNA: long non-coding RNA
miRNA: microRNA
mRNA: messenger
CSCs: cancer stem cells
ncRNAs: noncoding RNAs
CDK2: cyclin-dependent kinase 2
p21: cyclin-dependent kinase inhibitor 1
MBL: muscleblind
IRESs: Internal ribosomal entry sites
OPF: open reading frame
cUTRs: UTRs of ribo-circRNAs
RFP: ribosome footprinting
RCA: Rolling circle amplification
BCSCs: breast cancer stem cells
TCF3: transcription factor 3
TERT: telomerase reverse transcriptase
MET: mesenchymal-epithelial transition
UCHL1: ubiquitin C-terminal hydrolase L1
BACAE1: APP-cleaving enzyme 1
OCSCs: ovarian cancer stem cells
ECM: extracellular matrix
T-HaCaT cells: arsenite-transformed HaCaT cells
DAB2IP: DAB2 interacting protein
ZEB1: zinc finger E-box binding homeobox 1
LCSCS: laryngeal cancer stem cells
DC: dendritic cell
G-CSF: granulocyte colony-stimulating factor
LT-HSCs: long-term- hematopoietic stem cells
cGAS: Cyclic GMP-AMP.
